# Preliminary Animal Study on Bone Formation Ability of Commercialized Particle-Type Bone Graft with Increased Operability by Hydrogel

**DOI:** 10.3390/ma14164464

**Published:** 2021-08-09

**Authors:** So-Yeun Kim, You-Jin Lee, Won-Tak Cho, Su-Hyun Hwang, Soon-Chul Heo, Hyung-Joon Kim, Jung-Bo Huh

**Affiliations:** 1 Department of Prosthodontics, Kyungpook National University Dental Hospital, Daegu 41940, Korea; soyeunkim179@gmail.com; 2 Department of Prosthodontics, Dental and Life Sciences Institute, Education and Research Team for Life Science on Dentistry, School of Dentistry, Pusan National University, Yangsan 50612, Korea; nicejin17@naver.com (Y.-J.L.); joonetak@hanmail.net (W.-T.C.); hsh2942@hanmail.net (S.-H.H.); 3 Department of Oral Physiology, Periodontal Diseases Signaling Network Research Center, Dental and Life Science Institute, School of Dentistry, Pusan National University, Yangsan 50612, Korea; snchlheo@gmail.com

**Keywords:** bone regeneration, bone substitute, bovine-derived xenograft, hydrogel

## Abstract

The purpose of this study was to evaluate the bone-generating ability of a new bovine-derived xenograft (S1-XB) containing hydrogel. For control purposes, we used Bio-Oss and Bone-XB bovine-derived xenografts. S1-XB was produced by mixing Bone-XB and hydrogel. Cell proliferation and differentiation studies were performed to assess cytotoxicities and cell responses. For in vivo study, 8 mm-sized cranial defects were formed in 16 rats, and then the bone substitutes were transplanted into defect sites in the four study groups, that is, a Bio-Oss group, a Bone-XB group, an S1-XB group, and a control (all *n* = 4); in the control group defects were left empty. Eight weeks after surgery, new bone formation areas were measured histomorphometrically. In the cell study, extracts of Bio-Oss, Bone-XB, and S1-XB showed good results in terms of the osteogenic differentiation of human mesenchymal stem cells (hMSCs) and no cytotoxic reaction was evident. No significant difference was observed between mean new bone areas in the Bio-Oss (36.93 ± 4.27%), Bone-XB (35.07 ± 3.23%), and S1-XB (30.80 ± 6.41%) groups, but new bone area was significantly smaller in the control group (18.73 ± 5.59%) (*p* < 0.05). Bovine-derived bone graft material containing hydrogel (S1-XB) had a better cellular response and an osteogenic effect similar to Bio-Oss.

## 1. Introduction

Currently, various types of graft materials are used for guided bone regeneration (GBR). Graft materials are classified as autografts, allografts, or xenografts, which have different osteoblast adhesion, proliferation, and differentiation properties [1]. Autografts are considered ideal graft material as regards osteogenesis, osteoinduction, and osteoconduction [2], but they require an additional surgical site for bone harvesting, which increases patient discomfort and risks of complications [3,4]. For this reason, development is being actively conducted on other types of bone graft materials [5,6].

Synthetic bone material and xenograft have advantages in terms of supply and cost. Synthetic bone materials, including ceramic-based hydroxyl-apatite (HA) and tricalcium phosphate (β-TCP), but are limited in terms of biocompatibility, osteoconduction, and osteoinduction [7,8]. In addition, since synthetic bone materials are generally stronger than surrounding bone stress, shielding problems may occur [9]. Xenografts obtained from animals (e.g., bovine and porcine sourced) are similar to human bones [10], and because xenografts are biocompatible and have high porosities, they provide space for osteoinduction and osteoconduction [11,12]. Bovine-derived xenografts are the most widely used graft materials and have been reported on many occasions to produce good results [13,14].

Graft materials are made in the form of particles of various sizes and are applied using the conventional GBR technique by compaction in transplant areas and covering the compacted materials with a membrane. However, when defects are irregularly shaped or large, it is difficult to maintain particle-type bone graft materials stably, and prognoses are relatively poor [15]. In addition, particle-type bone graft materials do not compact well due to a lack of adhesion between particles, and thus, handling properties deteriorate during surgery, and sometimes graft particles are malpositioned or lost [16,17]. In order to solve these problems, several types of bone graft materials have recently appeared on the market. These products improve handling properties by preventing particle separation by interposing organic substances between bone graft particles [18].

Hyaluronic acid, glycerol, chitosan, and others have been used as carrier materials to increase the handling of bone graft powder [19]. Recently, moldable putties were developed to fill bone defects and are being widely used because they hold their shapes and are not dispersed by irrigation or blood [20]. In previous studies, various hydrogels were applied to bone grafts in combination with bioactive molecules or cells [21]. In particular, in a recent review article, it was reported that hydrogels improve the stabilities of graft materials in injection or graft sites [22], and in a recent study, alginate hydrogel was reported to improve bone graft stability during surgery [23].

The use of hydrogels for preparing bone grafts confers several advantages. Hydrogels have several potential benefits for bone repair because they have swollen network structures that can contain biologically active agents and excellent biocompatibility. Furthermore, the three-dimensional hydrophilic characteristics of hydrogels provide mechanical strength and nutritional environments suitable for endogenous cell growth. The hydrogel reduces inflammatory responses, has soft textures, and its viscoelastic properties increase operability [21,24,25]. It has also been reported that hydrogels promote the spreading, proliferation, and differentiation of mesenchymal stem cells [26]. The use of hydrogels by mixing with bone graft materials such as ceramics (hydroxyapatite, tricalcium phosphate) and bioglass particles has been studied [27].

Currently, many products for bone graft are being developed, and attempts to improve the operability of the widely used bovine-derived xenograft materials are continuing. The development of injectables, putties, cements, pastes, gels, etc. is attractive in the field of bone regeneration because of its formability and stability, and products with improved operability using various materials are on the market [28]. However, there are few studies evaluating the clinical application of hydrogel-added xenograft materials among various materials. The purpose of this study is to evaluate the clinical applicability of a commercially available xenograft material developed to be manipulated in a shape suitable for the defect area by applying particle xenograft materials and hydrogels at the cellular and small animal level.

## 2. Materials and Methods

### 2.1. Experimental Xenogenic Bone Substitutes

In this study, two commercial, powder-type bovine-derived bone grafts were used as experimental groups. Bio-Oss group (Bio-Oss^®^, Geistlich Pharma AG, Wolhusen, Switzerland) is the most widely used product in the market and is generally used as a positive control because of its considerable scientific basis. Bone-XB group (Bone-XB^®^, Medpark, Busan, Korea) is also derived from bovine cancellous bone, but its granule size is smaller and its Ca/P ratio is greater than that of Bio-Oss. S1-XB group (S1-XB^®^, Medpark, Busan, Korea) is prepared by mixing Bone-XB with hydrogel. The company did not disclose patent or specific manufacturing details. Specific porosity, Ca/P ratio, particle sizes, and manufacturers’ details are provided in Table 1 [16].

### 2.2. In Vitro Study

#### 2.2.1. Scanning Electron Microscope (SEM) Image Analysis

The surface morphology of the xenogenic bone substitutes was observed by scanning electron microscopy (SEM, Hitachi S3500N, Hitachi, Tokyo, Japan). Specimens were coated with Au using a sputter coater (SCD 005, BAL-TEC, Balzers, Liechtenstein), and SEM was conducted at an accelerating voltage of 15kV. Surface compositional analysis was performed by onboard energy-dispersive X-ray spectroscopy (EDX, Apollo X, Ametek EDAX, Mahwah, NJ, USA). EDX dot scans were performed three times for each group of specimens.

#### 2.2.2. Preparation of Extracts

We mixed 250 mg of each xenogeneic bone substitute (Bio-Oss, Bone-XB, and S1-XB) with 10 mL of alpha-modified Eagle’s medium (α-MEM; Welgene, Deagu, Korea) and incubated suspensions at 37 °C for 24 h. Control medium was prepared in the same manner without bone substitute. Suspensions were centrifuged at 1500 rpm for 5 min and filtered through 0.2 μm membranes (Sartorius, Göttingen, Germany).

#### 2.2.3. Culture of Human Bone Marrow Mesenchymal Stem Cells (hMSCs)

Human mesenchymal stem cells (hMSCs) were purchased from Lonza (Walkersville, MD, USA) and cultured in α-MEM supplemented with 10% fetal bovine serum (FBS; Gibco, Carlsbad, CA, USA), 100 U/mL penicillin, and 100 µg/mL streptomycin (Gibco^TM^, Thermo Fisher Scientific, Waltham, MA, USA), at 37 °C in a 5% CO_2_ atmosphere. Media were refreshed every other day, and the hMSCs used in all experiments were passaged less than seven times.

#### 2.2.4. Cell Viability and Proliferation Assay

hMSCs were seeded into 48-well plates (Nunc, Roskilde, Denmark) at a density of 10^4^ cells/well in medium containing 20% or 50% of the bone substitute extracts and cultured for 0, 1, 2, or 3 days. Cell proliferations were assessed by adding 20 µL of CCK-8 solution (Dojindo, Rockville, MD, USA). Absorbances were measured at 450 nm using an Opsys MR micro-plate reader (DYNEX Technologies Inc., Denkendorf, Germany). Cell viabilities were determined with 24 h absorbance data.

#### 2.2.5. Osteoblasts Differentiation and Alkaline Phosphatase (ALP) Activity Assay

hMSCs were seeded into a 48-well plate at a density of 10^4^ cells/well and cultured for 24 h. Osteoblast differentiation was induced by adding 10 mM of β-glycerophosphate and 50 µg/mL of ascorbic acid (Sigma-Aldrich, Milan, Italy) to α-MEM supplemented with 10% FBS. The medium was refreshed every other day. Bone substitute extracts constituted 50% of the total osteogenic induction medium. On day 12, alkaline phosphatase (ALP) activities were estimated using the ALP staining kit (86R-1KT, Sigma-Aldrich, Milan, Italy). Intensities of alkaline phosphatase staining were quantified by imaging culture plates (Nikon, Eclipse Ts2, Melville, NY, USA) and measuring integrated densities using the ImageJ program (version 1.52p, National Institutes of Health, Bethesda, MD, USA).

#### 2.2.6. Quantitative Real-Time Polymerase Chain Reaction (qPCR) Analysis

The qPCR analysis was performed to examine the expressions of osteogenic differentiation markers. Five days after osteogenic induction, total RNA was extracted from hMSCs using TRIzol (Life Technologies, Grand Island, NY, USA), and 2 µg of RNA was reverse transcribed using Superscript II (Invitrogen) (Gibco^TM^, Thermo Fisher Scientific, Waltham, MA, USA) according to the manufacturer’s instructions. For qPCR analysis, 50 ng of cDNA was mixed with SYBR Green PCR Master Mix (Applied Biosystems, Forster, CA, USA) and amplified for 40 cycles using an AB7500 unit (Applied Biosystems). Experiments were performed in triplicate, and the data were normalized versus β-actin mRNA. Besides GAPDH, beta-actin is one of the most widely used genes as an internal reference gene for quantitative real-time PCR. The stable expression of beta-actin has been validated in human mesenchymal stem cell as well as osteoblast [29,30]. The analysis was performed using the ∆(∆CT) method. The primer sequences used in qPCR were as follows: Runx2: F(5′-TGCTTTGGTCTTGAAATCACA-3′), R(5′-TCTTAGAACAAATTCTGCCCTTT-3′); ALP: F(5′-ATCCAGAATGTTCCACGGAGGCTT-3′), R(5′-AGACACATATGATGGCCGAGG); OCN: F(5′-CAGCGAGGTAGTGAAGAGAC-3′), R(5′-TGAAAGCCGATGTGGTCAG-3′); OSX: F(5′-TCCCTGCTTGAGGAG GAAG-3′), R(5′-AGTTGTTGAGTCCCGCAGAG-3′); OPN: F(5′-AGACACATATGATGGCCGAGG-3′), R(5′-GGCCTTGTATGCACCATTCAA-3′); β-actin: F(5′-ACTCTTCCAGCCTTCCTTCC-3′), R(5′-TGTTGGCGTACAGGTCTTTG-3′) (Table 2).

### 2.3. In Vivo Experiment

#### 2.3.1. Animals and Operative Procedures

**Figure 1:**
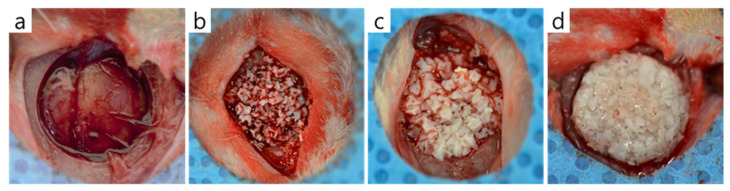
The bone graft materials applied to rat calvaria defects. (**a**) Control group, (**b**) Bio-Oss group, (**c**) Bone-XB group, and (**d**) S1-XB group.

The experimental protocol was approved beforehand by the IACUC (Institutional Animal Care and Use Committee), the Animal Experimental Ethics Committee of Pusan National University (PNU-2020-2618). Sixteen Sprague-Dawley rats (male, 13 weeks old, Koatech, Pyeongtaek, Korea) were used for in vivo experiments. Animals were acclimated to a pellet diet with free access to water in individual cages for 2 weeks. For surgery, general anesthesia was achieved by administering an intraperitoneal injection of avertin. In each animal, the skull was exposed through a sagittal incision using a #15 surgical blade, and a trephine bur was used to make a circular defect of diameter 8 mm under saline irrigation. Bone graft materials were applied to defects according to the manufacturer’s instructions (Figure 1), and surgery was completed by suturing periosteum and skin with 3-0 Vicryl sutures without a membrane.

Eight weeks after surgery, rats were sacrificed by CO_2_ inhalation. Tissue samples, which included peri-defect samples, were carefully secured and fixed in 10% neutral buffered formalin (Sigma-Aldrich) for 2 weeks.

#### 2.3.2. Histologic Analysis

**Figure 2:**
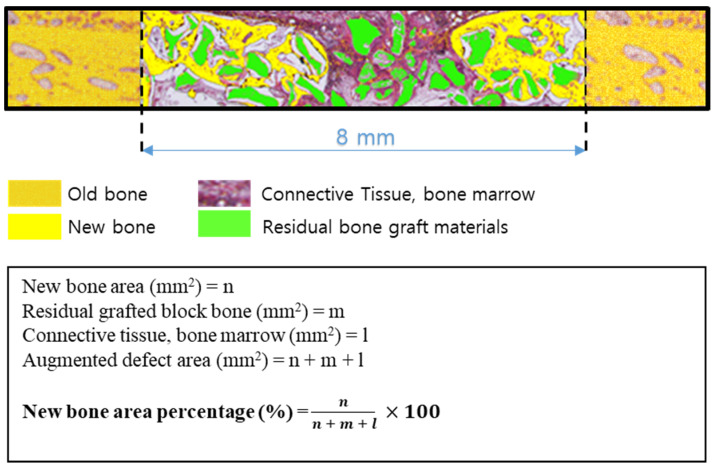
Schematic of histometric analysis.

Specimens were decalcified using decalcifying Solution (Calci-Clear Rapid^®^, National Diagnostics, Atlanta, GA, USA) and dehydrated in an alcohol series (70%, 80%, 90%, and 100%) before they were embedded in paraffin. After that, the paraffin block was sectioned 3–4 μm thickness using a microtome(Leica^®^ RM2255, Leica Biosystems Inc., Buffalo Grove, IL, USA). Slides were stained with hematoxylin-eosin and Masson’s trichrome and imaged under an optical microscope (Olympus BX51, Olympus Co., Ltd., Tokyo, Japan). New bone areas (%) were determined using an image analysis program (iSolution, IMT, Vancouver, Canada) (Figure 2).

#### 2.3.3. Statistical Analysis

The analyses were performed using SPSS ver. 25.0 (SPSS, Chicago, IL, USA). In vitro data were obtained from at least three independent experiments conducted in triplicate. Results of multiple observations are presented as means ± SEMs. The significances of intergroup differences were determined by one-way or two-way ANOVA followed by the Bonferroni post hoc test. In vivo results were analyzed using the Kruskal-Wallis test followed by the Mann-Whitney U post hoc test. Statistical significance was accepted for *p* values < 0.05.

## 3. Results

### 3.1. In Vitro Findings

#### 3.1.1. Scanning Electron Microscope Surface Analysis

**Figure 3:**
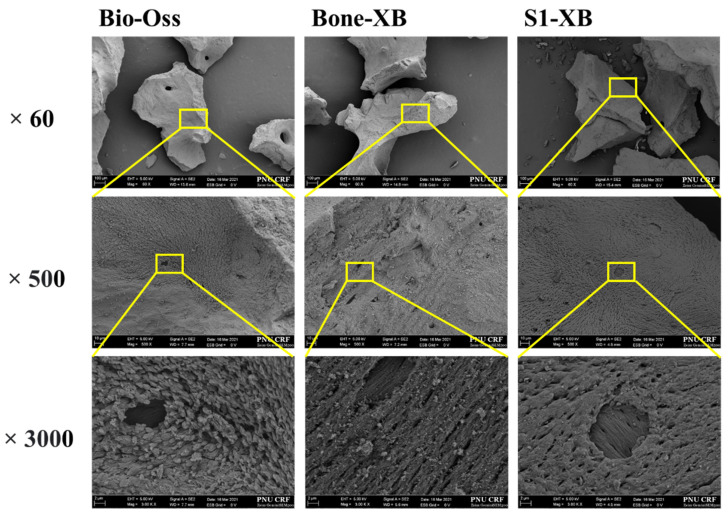
Scanning electron microscope (SEM) images of each group. [Original magnification: ×60, ×500, ×3000.

Images were taken at ×60, ×500, and ×3000 magnification to investigate the surface morphologies of bone graft materials. Similar particle sizes and macro-porous structures were observed for Bio-Oss, Bone-XB, and S1-XB. In S1-XB, distinct hydrogel layers were not visible on the SEM image (Figure 3).

#### 3.1.2. Energy-Dispersive X-ray Spectroscopy (EDX) Findings

The elements in bone graft materials and Ca/P ratios were determined by EDX (Table 3). Ca/P ratios were similar for Bio-Oss and Bone-XP, the ratio was slightly lower for S1-XB. For all bone substitutes, the stoichiometric ratio of HA was less than 1.67. In addition, S1-XB had higher C and O contents than Bio-Oss or Bone-XB, which attributed to the hydrogel.

#### 3.1.3. CCK-8 Assays of Cell Viability and Proliferation

**Figure 4:**
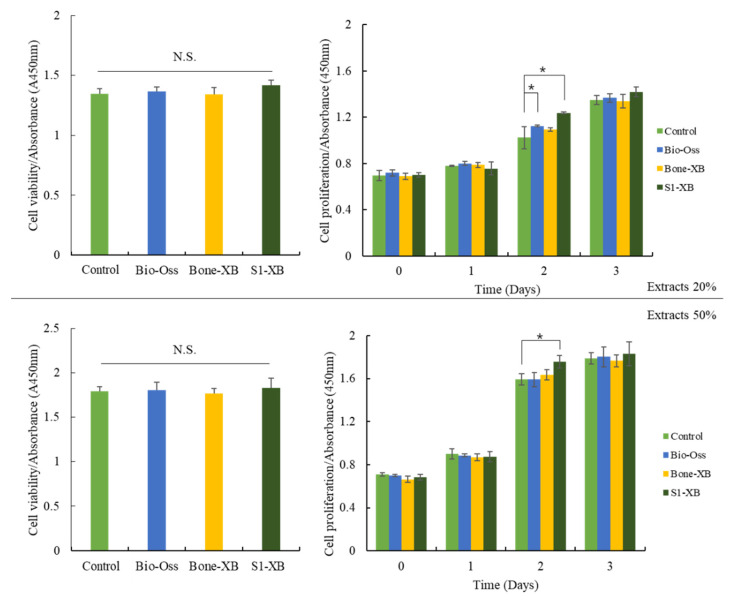
Cell viability and proliferation of human mesenchymal stem cells (hMSCs) exposed to the different extracts. The letter “*” indicates significant differences (*p* < 0.05); N.S.—No significant.

Liquid extraction method has been recommended as a standard procedure in testing cytotoxicity of biocompatible materials by the ISO 10993. After treating hMSCs with extracts of bone substitutes, cell viability and proliferation were investigated using the CCK-8 assay. Extracts were collected by incubating Bio-Oss, Bone-XB, or S1-XB for 24 h. No significant intergroup viability differences were observed after incubation for 24 h with 20% or 50% extracts of the bone substitute versus non-treated controls. hMSCs proliferation was measured every 24 h for 72 h after supplementation with 20% or 50% extracts. Supplementation with 20% extracts derived from Bio-Oss significantly increased hMSCs proliferation at 48 h. Notably, supplementation with 20% and 50% extracts derived from S1-XB increased hMSCs proliferation most at 48 h (Figure 4).

#### 3.1.4. Alkaline Phosphatase (ALP) Staining for Osteogenic Differentiation Analysis

**Figure 5:**
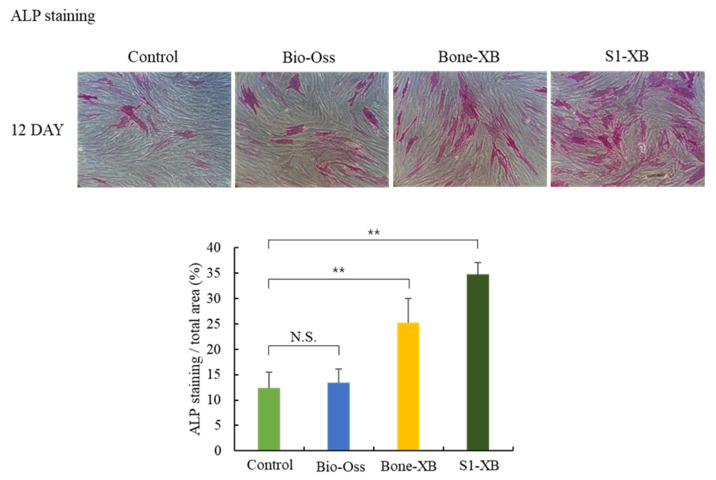
Cell osteogenic differentiation assay (** *p* < 0.01). The letter “**” indicates significant differences (*p* < 0.05); N.S.—No significant.

Since we could not find significant differences between 20% and 50% extracts treatment on hMSCs proliferation at the final time point in our system, only 50% extracts were used in the subsequent experiments. To determine the effects of bone substitutes on osteogenic differentiation, ALP staining was conducted 12 days after the induction of osteogenic differentiation by culturing hMSCs in the presence of 50% extracts. No obvious differences were observed between the ALP positive areas of cells treated with Bio-Oss extracts and non-treated controls. However, treatments with Bone-XB and S1-XB extracts significantly increased ALP positive areas versus controls, and the greatest increase was observed for cells treated with S1-XB extracts (Figure 5).

#### 3.1.5. Quantitative Real-Time Polymerase Chain Reaction (qPCR) for Osteogenic Differentiation Analysis

**Figure 6:**
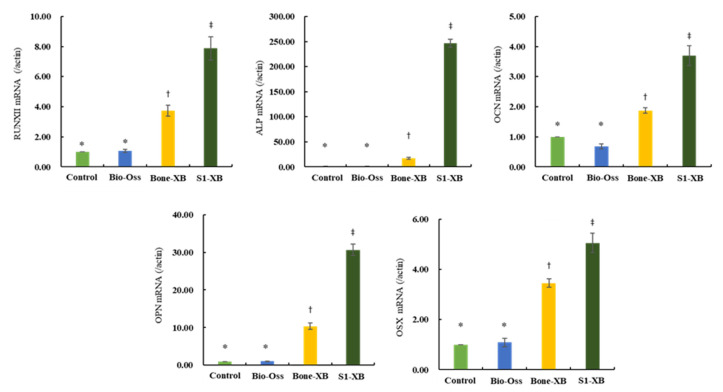
The effects of the three bone substitute extracts on osteogenesis-related genes. hMSCs were cultured in osteogenic induction media supplemented with 50% extracts for 12 days. The mRNA expressions of osteogenic differentiation-related genes, that is, Runx2, OSX, ALP, OCN, and OPN were determined by qPCR. Normalization was performed versus β-actin expression. “*”, “†”, “‡” Different letters indicate significant differences (*p* < 0.05).

To evaluate the effects of the three bone substitutes on osteogenic differentiation further, qPCR was performed to examine the expressions of osteogenesis-related genes. Consistent with ALP staining results, supplementation of extracts derived from Bio-Oss did not induce any osteogenesis-related genes versus non-treated controls. However, supplementation of extracts derived from Bone-XB and S1-XB greatly induced expressions of the key osteogenic transcription factors Runx2 and OSX and those of genes involved in osteoblast differentiation and matrix mineralization; ALP, OCN, and OPN (Figure 6).

### 3.2. In Vivo Findings

#### 3.2.1. Clinical Findings

All rats survived during the experimental periods, and no side effects such as inflammation or specimen exposure were observed.

#### 3.2.2. Histologic Findings

**Figure 7:**
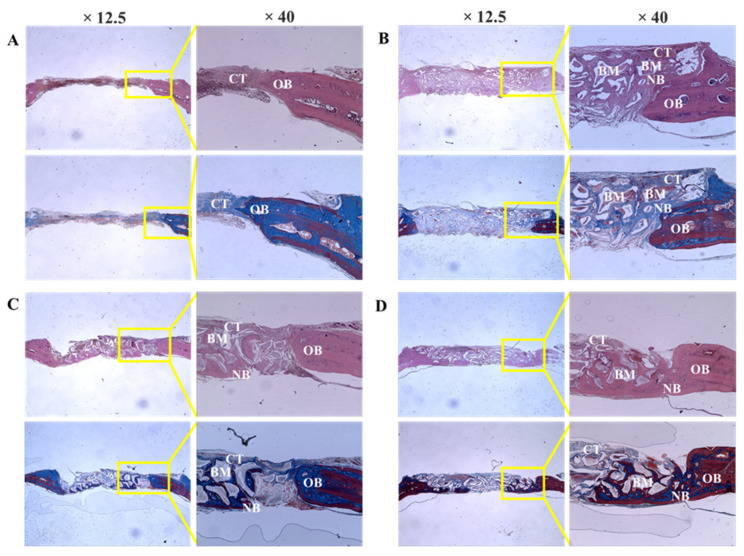
Histologic sections of defect sites at 8 weeks after surgery. (**A**) Control, (**B**) Bio-Oss, (**C**) Bone-XB, (**D**) S1-XB. Note: NB; new bone, CT; connective tissue, GM; graft materials, OB; old bone.

Bio-Oss, Bone-XB, and S1-XB bone substitutes were retained stably at defect sites, and no inflammatory cells or unexpected reactions were observed (Figure 7).

#### 3.2.3. Histomorphometric Findings

**Figure 8:**
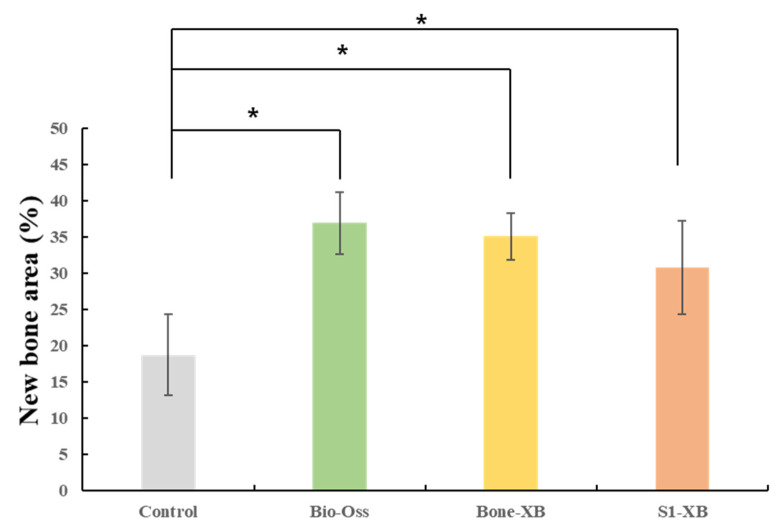
Percentages of new bone area within areas of interest (AOIs). The letter “*” indicates significant differences (*p* < 0.05).

New bone area results are shown in Table 4 and Figure 8. New bone area was significantly less in the control group than in the Bio-Oss, Bone-XB, or S1-XB groups, but no significant difference was observed between the three bone graft materials (*p* > 0.05).

## 4. Discussion

In this study, we compared three commercially available xenograft materials that have been officially confirmed to be safe. The first one, Bio-Oss is well-known for its effectiveness, safety, bone formation quality and quantity, and high success rate [31,32,33]. The second one, Bone-XB is a new bovine bone graft material developed in Korea. It has a particle size of 0.2–1.0 mm and a macro/micropore structure similar to Bio-Oss. The third one, S1-XB is a product from the same manufacturer as Bone-XB, a mixture of the xenograft particle and hydroxypropyl methylcellulose that is the polymer making the hydrogel. After the xenograft particle adsorbed with osteogenic protein and hydroxypropyl methyl cellulose powder are mixed and stirred to obtain a viscous gel, it is prepared under vacuum and freeze-drying conditions. Cause of the viscous condition, S1-XB had a putty-like consistency that allowed it to be shaped easily by hand, and it also showed an ability to retain its shape. In Figure 1b,c, the particles of the material are together in the defect, but the particles are seen separately one by one. The graft material in Figure 1d looks like a single, round lump with a rough surface.

The electron microscopy (SEM) images of Bio-Oss, Bone-XB, and S1-XB groups showed similar pore morphologies, presumably because they are all derived from bovine bone. Macro pores contribute to the formation of blood vessels, whereas micro pores contribute to osteoblast adhesion, and thus, Bio-Oss and Bone-XB can be considered to provide environmentally similar osteoblast scaffolds [34]. In the S1-XB group, the hydrogel coating layer was not well-visualized by SEM, which implied it was evenly and thinly distributed. a is useful for studying the degree of bone mineralization [35], and the theoretical value (1.67) of stoichiometric hydroxyapatite, the main component of human bone, and the Ca/P ratio of the three bone substitutes were different. Bone-XB was 1.550, Bio-Oss was 1.526, and S1-XB was 1.434, all smaller than 1.67 [36,37]. In the previous study, when the Ca/P ratio was 2.0 or higher, it decreased osteoblast viability after 72 h; in contrast, when the Ca/P ratio was less than 2.0, it optimized osteoblast viability and promoted osteoblast alkaline phosphatase activity after 72 h. In addition, although HA with a Ca/P ratio of 1.67 has excellent biocompatibility, it is known to interrupt bone re-generation due to slow absorption in the body. However, TCP with Ca/P 1.5, a commercially available bone graft material, has high biodegradability and is mixed with HA in a certain ratio to compensate for each disadvantage. The materials used in this study showed results close to TCP with Ca/P of 1.4 to 1.5. Compared to Bio-Oss and Bone-XB, the C and O levels were much higher in S1-XB, and thus, Ca and P levels were reduced, presumably due to the presence of the hydrogel [24]. The hydrogel is made of various polymers, and since there are many C and O in the polymer, these results were expected to be shown in S1-XB [38].

In previous studies, Bio-Oss was reported to have a positive effect on the osteoblast differentiation of hMSCs [39]. In this study, S1-XB and Bone-XB were compared with Bio-Oss. In the cell study, the toxicity evaluation was performed by measured CCK-8 assay, areas of differentiated osteoblastic cells were compared by ALP staining, and levels of bone remodeling-related mRNAs were assessed by real-time PCR. All three bone substitutes had viabilities and cell proliferations similar to those of non-treated controls. Degrees of osteoblast differentiation followed the order S1-XB > Bone-XB > Bio-Oss and were similar for Bio-Oss and non-treated controls group but higher for S1-XB and Bone-XB than controls. ALP activity was investigated by real-time PCR as a surrogate of osteoblast activity because ALP is a marker of the change from pre- to mature osteoblasts. Our results show that S1-XB extract significantly promoted osteoblast differentiation. In addition, levels of the transcription factors of osteoblast differentiation Runx2 and osterix (OSX) were measured. OSX and Runx2 levels were significantly higher for S1-XB followed by Bone-XB. Extracellular matrix proteins, osteopontin (OPN) and osteocalcin (OCN), which are expressed during the formation of osteoblast or woven bone formation, were also assessed by PCR. The results showed expressions were significantly higher for S1-XB than Bone-XB and significantly higher for Bone-XB than Bio-Oss [40,41,42]. The CCK-8 assay revealed that the extracts from Bio-Oss, Bone-XB, and S1-XB showed no significant cytotoxic effect on hMSCs viability under these experimental conditions. The modest proliferation of hMSC was observed in response to supplementation with Bio-Oss and S1-XB at 48 h, but no detectable differences at 72 h. Based on these results, it appears that the osteoinductive effects of bone graft extracts on hMSCs are mainly derived by inducing osteogic differentiation capacity, not the stimulation of proliferation.

Histological analysis showed osteoblasts were distributed around transplanted xenografts in the Bio-Oss, Bone-XB, and S1-XB groups, indicating that all three xenografts used in this study had good bone conductivity [43]. Histomorphometric analysis showed new bone areas were not significantly different for the three bone substitutes, though all had significantly larger new bone areas than in the control group. Summarizing, S1-XB produced better results in cell studies and similar results in animal studies than Bio-Oss or Bone-XB, which shows S1-XB is biocompatible and osteoblast differentiation characteristics similar to that of Bio-Oss.

A wide variety of products are now in production, requiring clinicians to evaluate new products and validate their clinical applicability. It is meaningful that a product with a new function, such as S1-XB, has no significant difference in animal test results from existing products that are acknowledged and used. The main problems of previous bone graft material particles were manipulation, transportation, and molding [44,45]. The original particle-type bone graft material is less stable due to its intrinsic physical properties, so a space-maintaining barrier such as a membrane must be used [46]. S1-XB with the addition of hydrogel was not inferior to the existing materials in terms of bone formation ability in this study, even though no membrane was used. Products with similar bone formation performance and superior operability are valuable in clinical practice.

There are several limitations in the current study that are worth considering. First limitation, since this study was designed as a pilot, the number of animals is limited, so results from animal studies may be inferior to cell studies. Second limitation, as the graft materials have cohesiveness, we did not cover the graft areas with collagen membrane, although we doubt whether the absence of a membrane caused xenograft losses or displacements. The barrier membrane can prevent bone graft material loss by ensuring the stability of the bone graft material [47]. Third limitation, we have not investigated whether the increased cohesiveness of S1-XB affects passages and porosity of graft materials for new bone growth [48]. Further research is required on these topics. Despite the limitations of this study, the surface properties, cell activities, and bone regeneration ability of S1-XB were not inferior to Bio-Oss, and it was increased operability by hydrogel, which indicated its suitability as a bone graft material. Further research is required on these materials and topics.

## 5. Conclusions

Within the limitations of this preliminary study, the osteogenic capacity and biocompatibility of S1-XB, a commercially available particle-type bone graft with increased manipulability by hydrogel, were not inferior to those of currently commercially available bovine-derived xenograft materials, and showed favorable operability clinically.

## Figures and Tables

**Table 1:** Characteristics of bone graft materials.

Product Name	Type	Derivation	Granule Size (µm)	Porosity (%)	Ca/P (%)	Manufacturer
Bio-Oss^®^	Powder	Bovine cancellous	250–1000	70.5	1.54	Geistlich Pharma AG, Wolhusen, Switzerland
Bone-XB^®^	Powder	Bovine cancellous	200–1000	70.20	1.7063	Medpark, Busan, Korea
S1-XB^®^	Powder	Bovine cancellous/hydrogel	200–1000	70.20	1.7063	Medpark, Busan, Korea

**Table 2:** Primer sequences used for real-time PCR.

Target Genes	Sequences
Runx2	F: 5′-TGCTTTGGTCTTGAAATCACA-3′
	R: 5′-TCTTAGAACAAATTCTGCCCTTT-3′
ALP	F: 5′-ATCCAGAATGTTCCACGGAGGCTT-3
	R: 5′-AGACACATATGATGGCCGAGG
OCN	F: 5′-CAGCGAGGTAGTGAAGAGAC-3′
	R: 5′-TGAAAGCCGATGTGGTCAG-3′
OSX	F: 5′-TCCCTGCTTGAGGAG GAAG-3′
	R: 5′-AGTTGTTGAGTCCCGCAGAG-3′
OPN	F: 5′-AGACACATATGATGGCCGAGG-3′
	R: 5′-GGCCTTGTATGCACCATTCAA-3′
β-actin	F: 5′-ACTCTTCCAGCCTTCCTTCC-3′
	R: 5′-TGTTGGCGTACAGGTCTTTG-3′

**Table 3:** EDS results of the bone substitutes (Atomic %; Mean ± SD).

Elements	Bio-Oss	Bone-XB	S1-XB
C	2.947 ± 1.034	2.29 ± 0.611	6.837 ± 6.433
O	28.19 ± 9.45	27.047 ± 6.339	34.78 ± 5.699
P	35.91 ± 1.192	37.04 ± 2.581	33.79 ± 7.053
Ca	54.78 ± 4.996	57.40 ± 3.789	48.44 ± 8.954
Ca/P	1.526	1.550	1.434

**Table 4:** New bone area within areas of interest (AOIs).

	Group	Mean	SD	*p*-Value
New bone area (%)	Control	18.73	5.59	0.026 *
	Bio-Oss	36.93	4.27	
	Bone-XB	35.07	3.23	
	S1-XB	30.80	6.41	

The letter “*” indicates significant differences (*p* < 0.05).

## Data Availability

Data sharing not applicable.
